# Low cost, portable, 3D printable tissue precision slicer

**DOI:** 10.1016/j.ohx.2024.e00611

**Published:** 2024-11-22

**Authors:** Beatriz Martinez-Martin, Isabella Lambros, Lukas Nuesslein, Yubing Sun

**Affiliations:** a Molecular and Cellular Biology Graduate Program, University of Massachusetts Amherst, Amherst, MA 01003, United States; b Department of Mechanical and Industrial Engineering, University of Massachusetts Amherst, Amherst, MA 01003, United States; c Department of Biomedical Engineering, University of Massachusetts Amherst, Amherst, MA 01003, United States

**Keywords:** Tissue slices, Microtomy, Organoids, Low-cost

## Abstract

Slicing tissue samples into thin pieces is commonly used in histology analysis and more recently for organotypic culture when tissue samples are sliced alive. Currently available devices for slicing tissue samples are either designed for fixed tissue samples at low cryogenic temperatures (*e.g.*, Cryostats), or bulky and expensive (*e.g.*, vibratome), preventing them from routine lab usage. Here we report a cost-effective device designed to section live tissues for subsequent culture. This device consists of components crafted from 3D-printed Nylon-12- a material suitable for autoclaving to ensure sterility. Its small footprint enhances portability, allowing for convenient placement within a biosafety cabinet for an added layer of sterility assurance. Using human pluripotent stem cells derived brain organoids as an example, we demonstrated that the device both precisely and accurately makes slices. We further validate its suitability for long-term culture by extended tissue culture following slicing. Our results indicate that brain organoid slices are viable and show improved proliferation rate compared with unsliced organoids.

## Graphical abstract

**Figure:**
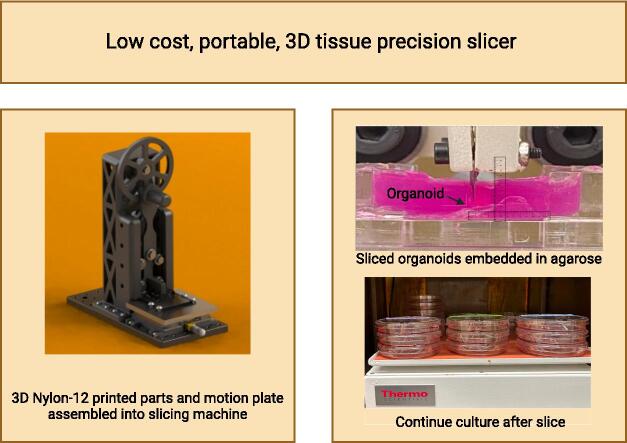

## 1 Specifications table

**Table:** 

Hardware name	Live Tissue Precision Slicer
Subject area	• Engineering and materials science • Neuroscience • Biological sciences
Hardware type	• Imaging tools • Biological sample handling and preparation • Mechanical engineering and materials science
Closest commercial analog	• Vibratome • Cryostat
Open source license	CC-BY-NC 4.0
Cost of hardware	∼$853.21 USD
Source file repository	https://doi.org/10.17632/p3npz8wkny.2

## 2 Hardware in context

Tissue slicing is a widely employed technique in laboratories for diverse applications such as immunohistochemistry, electrophysiological analysis, in situ hybridization, and electron microscopy [1], [2], [3]*.* Recently, this method has been employed in the realm of live tissues such as organoids. Slicing organoids addresses the diffusion limits of nutrient and oxygen, and thus enables extended culture for improved viability and functional maturation [4]. For brain organoids, the sliced organoids have improved adhesion to planar multielectrode arrays, making it ideal for electrophysiological assessments [5]. Researchers typically utilize a microtome within a cryostat to section fixed tissue samples. However, this traditional approach relies on a biocompatible adhesive that solidifies only at extremely low temperatures, rendering it unsuitable for live tissue samples.

Considering the vulnerability of living tissues to such low temperatures, an alternative cutting method becomes imperative. Vibrating microtomes, or vibratomes, offer a solution for slicing live tissue [1]; however, they can be prohibitively expensive. On the more cost-effective and basic side, vibratomes are available from $5,000-$15,000 USD and more advanced models with additional features range from $20,000-$50,000 USD. Typical vibratome systems occupy significant spaces in a biosafety cabinet (BSC), and it is inconvenient to thoroughly sterilize the equipment given the complex geometry and delicate electrical components. Thus, a simple, cost-effective, and robust slicer is needed particularly for live-tissue slice culture.

This hardware was developed with three primary objectives: 1) **Cost effectiveness:** Providing an economical option for tissue slicing applicable to both live and fixed tissues; 2) **Optimization for live tissue:** Featuring a small form factor for compatibility with a BSC and standard sterilization practises; 3) **Precision and consistency**: reaching an accuracy of 0.25 µm for slice thickness without sacrificing cell viability. In this innovative design, organoids are suspended in agarose and secured within a biocompatible acrylic box, eliminating the need for the repeated purchase of adhesive material as the box is reusable. Here we demonstrate the feasibility of using this device for cutting live brain organoids. With some modifications, this device can be applied broadly for slicing multiple types of live or fixed tissues.

## 3 Hardware description

**Fig. 1:**
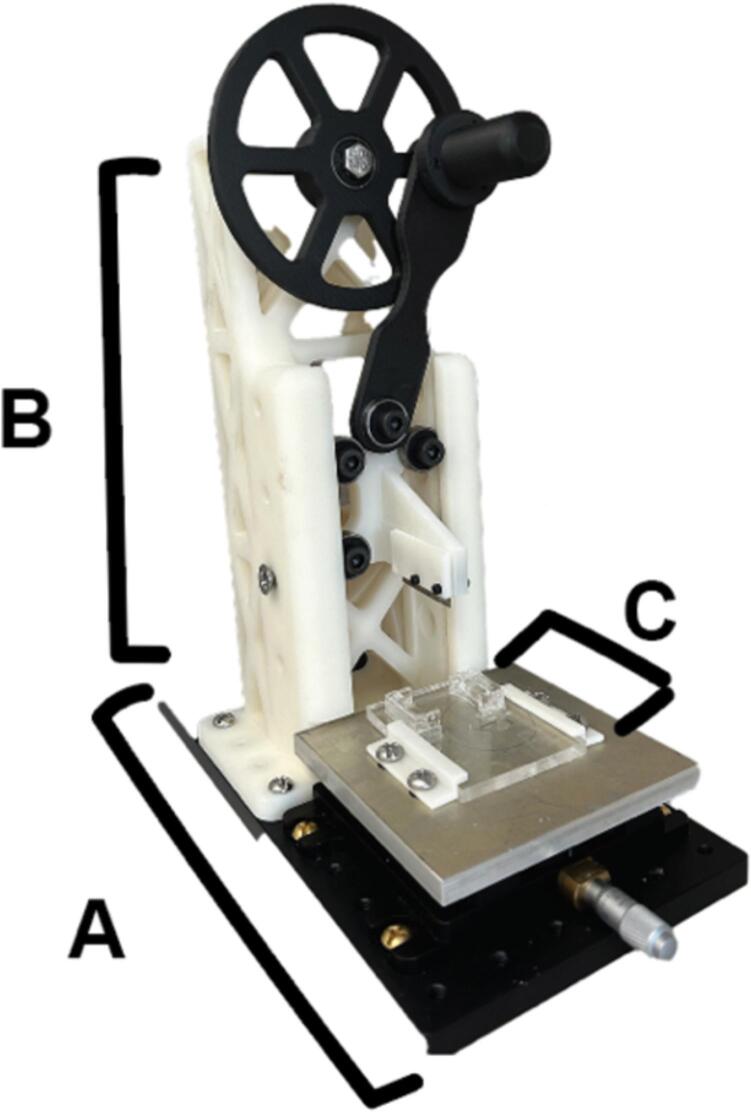
A photograph of the live tissue precision slicer. The device is mainly composed of a base with a motion plate (A), an arm with cutting blades (B), and a box (C) to hold agarose gel embedded tissues.

In its simplest form, this equipment is a highly precision slicing machine specifically designed to slice live tissue although it can be adapted for slicing fixed samples as well. The hardware comprises fundamental screws and bearings readily available at a hardware store or through online machining vendors. The machine is structured around three key components: the base, the arm, and the box (Fig. 1). The machine's base functions as a motion stage, allowing for adjustments to achieve various cut sizes. The machine’s arm is constructed from 3D printed Nylon-12 pieces that seamlessly interlock with screws and bearings, facilitating an intuitive and swift assembly process using only wrenches and screwdrivers. The machine’s box is composed of acrylic, and this is where the sample will be mounted to slice while embedded in agarose. Operating as a fully manual system, this machine enables easy customization of cuts, as well as overall hardware adjustments and optimizations tailored to your specific processes. Compared to a vibratome or a cryostat, this machine is significantly more cost effective and allows for live tissue slicing followed by long-term culturing. While varying by brand, vibratomes typically weigh around 15 kg, and cryostats weigh approximately 193 kg. In comparison, our machine weighs 7 kg, making it significantly smaller and more portable. The time required to section an entire organoid depends on its size and the desired slice thickness. For instance, once mounted, slicing at 300 µm using a vibratome takes approximately 30 min, and a cryostat takes approximately 20 min, whereas our device only requires about 1 min per slice. Our slicer offers a range of slice thicknesses from 250 µm to 1 mm, providing a comparable resolution to commercially available devices. Our data show that this slicer robustly makes quick and precise cuts to small tissue samples (less than 1 mm) with easily adjustable cut size.

***Design files***.

## 4 Design files summary

**Table:** 

**Design file name**	**File type**	**Open source license**	**Location of the file**
Acrylic_Box.SLDASM	CAD file	CC-BY-NC 4.0	https://doi.org/10.17632/p3npz8wkny.2
Acrylic_Box_and_Shell.SLDASM	CAD file	CC-BY-NC 4.0	https://doi.org/10.17632/p3npz8wkny.2
Acrylic_Shell.SLDASM	CAD file	CC-BY-NC 4.0	https://doi.org/10.17632/p3npz8wkny.2
Arm_Base.SLDPRT	CAD file	CC-BY-NC 4.0	https://doi.org/10.17632/p3npz8wkny.2
Crank.SLDPRT	CAD file	CC-BY-NC 4.0	https://doi.org/10.17632/p3npz8wkny.2
Large_Insert.SLDPRT	CAD file	CC-BY-NC 4.0	https://doi.org/10.17632/p3npz8wkny.2
Lever.SLDPRT	CAD file	CC-BY-NC 4.0	https://doi.org/10.17632/p3npz8wkny.2
Pin_Wall.SLDPRT	CAD file	CC-BY-NC 4.0	https://doi.org/10.17632/p3npz8wkny.2
Precision_Slicer.SLDASM	CAD file	CC-BY-NC 4.0	https://doi.org/10.17632/p3npz8wkny.2
Razor_Fastener.SLDPRT	CAD file	CC-BY-NC 4.0	https://doi.org/10.17632/p3npz8wkny.2
Razor_Holder.SLDPRT	CAD file	CC-BY-NC 4.0	https://doi.org/10.17632/p3npz8wkny.2
Razor_Holder_Assembly.SLDASM	CAD file	CC-BY-NC 4.0	https://doi.org/10.17632/p3npz8wkny.2
Shelf.SLDPRT	CAD file	CC-BY-NC 4.0	https://doi.org/10.17632/p3npz8wkny.2
Slotted_Wall.SLDPRT	CAD file	CC-BY-NC 4.0	https://doi.org/10.17632/p3npz8wkny.2
Small_Insert.SLDPRT	CAD file	CC-BY-NC 4.0	https://doi.org/10.17632/p3npz8wkny.2
Wheel.SLDPRT	CAD file	CC-BY-NC 4.0	https://doi.org/10.17632/p3npz8wkny.2

## 5 Bill of materials summary

**Table:** 

**Component**	**Number**	**Cost per unit-USD**	**Total cost- USD**	**Source of materials**	**Material type**
M8x1.25 Mm nut	pkg. 100	$9.67	$9.67	https://mcmaster.com	Steel
M8x1.25 Mm thread, 150 Mm long bolt	pkg. 5	$9.52	$9.52	https://mcmaster.com	Steel
M8x1.25 Mm thread,130 Mm long bolt	1	$3.50	$3.50	https://mcmaster.com	Steel
M8x1Mm thread, 30 Mm long bolt	pkg. 5	$9.20	$9.20	https://mcmaster.com	Alloy Steel
M2.5x0.45 Mm thread nut	pkg. 100	$4.19	$4.19	https://mcmaster.com	Alloy Steel
M2.5x 0.45 Mm thread, 15 Mm long bolt	pkg. 5	$16.82	$16.82	https://mcmaster.com	Alloy Steel
M2.5x0.45 Mm thread, 10 Mm long bolt	pkg. 100	$6.54	$6.54	https://mcmaster.com	Steel
M6x1Mm thread, 10 Mm long bolt	pkg. 100	$14.57	$14.57	https://mcmaster.com	Alloy Steel
M7x1Mm thread, 10 Mm long bolt	pkg. 25	$6.07	$6.07	https://mcmaster.com	Steel
8x22x7mm rolling bearing	pkg. 100	$18.79	$18.79	https://amazon.com	Steel
¼’’ Acrylic sheet	1	$24.09	$24.09	https://interstateplastics.com	Acrylic
Two-axis linear translation stage with rotating platform	1	$730.22	$730.22	https://thorlabs.com	Other
PA 2200*	1,379.57 cm^3^	$0.09/cc	$124.16	https://eos.info	Powder

*Note that PA 2200 was printed in-house, and the costs provided reflect the rates charged by the AddFab facilities for the material and their services.

## 6 Build Instructions

**Construction requires screw drivers and wrenches of various sizes. Ensure you have the correct part sizes and tools to prevent potential challenges and enable smooth, efficient device assembly.**

**Printing Information**

The parts were printed using the selective laser sintering (SLS) process on an EOS P110 machine. The material is BASF PA11 powder, printed with a bed temperature of 185C. Parts were depowdered, blasted with crushed glass abrasive, and water rinsed. The process is similar to that of commercial 3D printing service bureaus who use nylon-11 in an SLS process.

### 6.1 Base

**Fig. 2:**
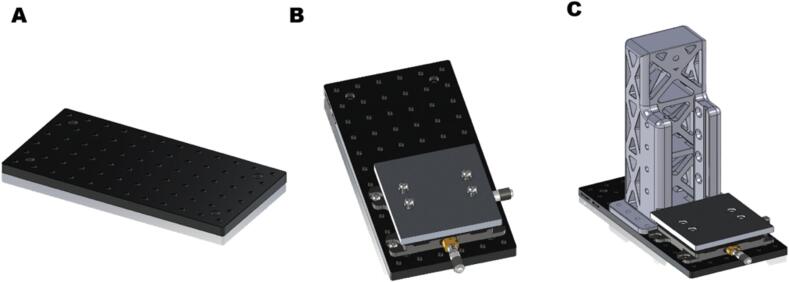
CAD models for Base component (A) Base with motion plate, (B) Fixture plate with attached motion plate to, and (C) Fixture plate with attached motion plate and arm base.

Using a screwdriver, attach the motion stage to the fixture plate with four ¼ in-20 x ¼ in screws and screw in four ¼ in-20 x 5/8 in screws. Attach two shelves to the motion stage using four ¼ in-20 x 5/8 in screws as shown below (Fig. 2**A-B**). Fasten the arm base to the fixture plate using eight ¼ in-20 x 5/8 in screws (Fig. 2**C**).

### 6.2 Arm

*Safety tip: It is recommended to wear cut resistant gloves when handling the sharp blade during assembly and operation

**Fig. 3:**
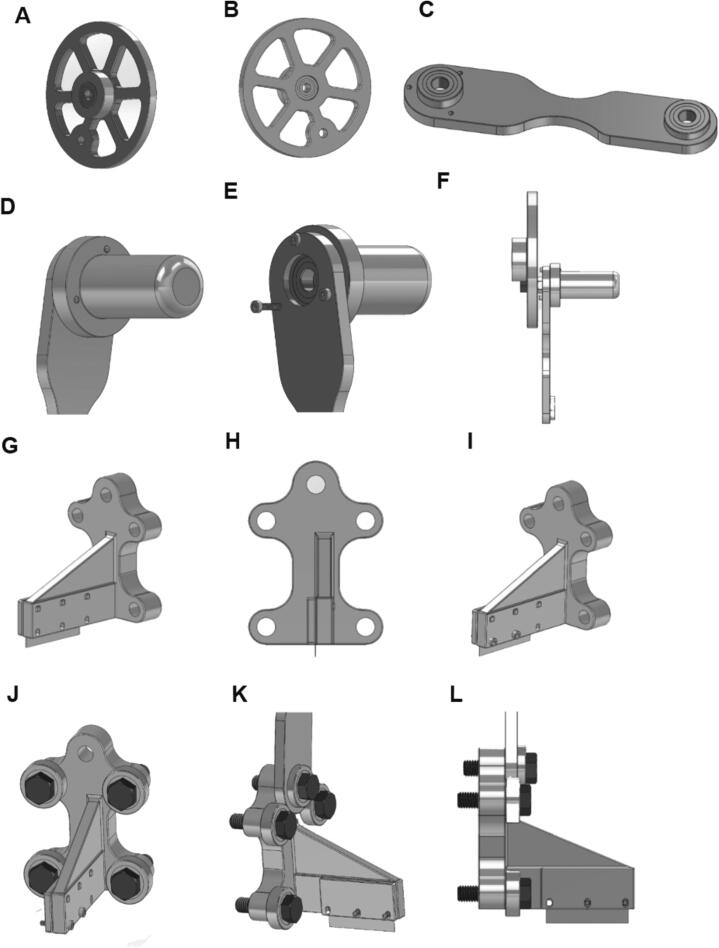
CAD models for arm components. (A-B) Wheel with bearings; (C) Lever with bearings; (D-E) Lever with crank; (F) Lever and crank attached to wheel; (G-I) Razor holder and fastener with razor; (J) Razor holder with screws and bearings; (K-L) Razor holder attached to lever.

5.2.1. Push two bearings into the wheel (Fig. 3**A-B**).

5.2.2. Push two bearings into the lever (Fig. 3**C**).

5.2.3. Align the lever with the crank and screw three M2.5 x 14 mm screws through both (Fig. 3**D-E**).

5.2.4. Screw an M8x 30 mm screw through the wheel, an M8 nut and the larger side of the lever.

(Fig. 3**F)**.

5.2.5 Align the razor’s holes with the three small holes on the razor holder and align the razor cover with the three-square slots (Fig. 3**G-H**).

5.2.6 Fasten the razor to the razor holder and razor cover using two M2.5 x .45 x 14 mm screws and two M2.5 nuts on the outer holes (Fig. 3**I**).

5.2.7. Place four M8 x 1.25 x 30 mm screws through four bearings and slide each of these into the front of the razor holder (Fig. 3**J**).

5.2.8. Put an M8 x 1.25 x 30 mm screw through the smaller side of the lever and then through the top of the razor holder (Fig. 3 **K-L**).

5.2.9. Slide the razor holder into the arm base slots.

5.2.10.Screw the M8 x 1.25 x 130 mm screw into the arm base so the end is just aligned with the wheel bearing outside face.

### 6.3 Box

**Fig. 4:**
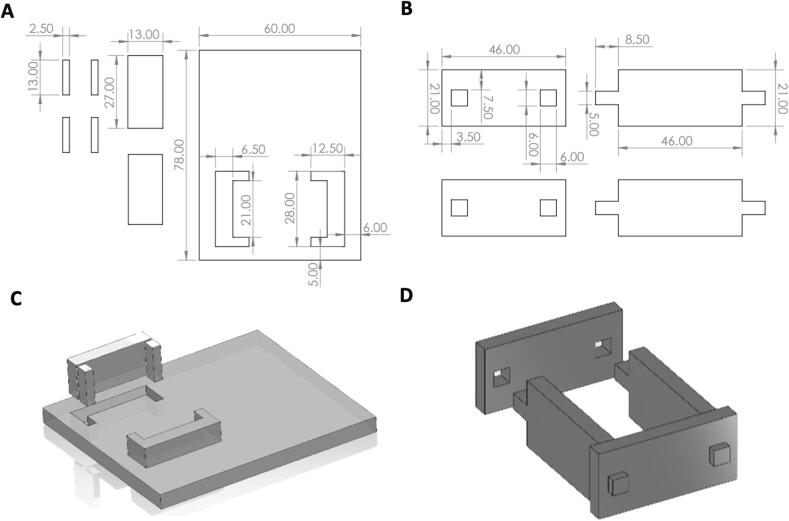
Acrylic box and box shell (A) Schematic of acrylic box (B) Schematic of acrylic box shell (C) Box (D) Acrylic box shell.

Cut out the schematic of the box using a laser cutter and ¼’’ acrylic. Assemble the acrylic box and shell by connecting the slotted pieces, as illustrated in Fig. 4. To bond the box together, apply polydimethylsiloxane (PDMS, Sylgard 184 10:1 (w/w) base: curing agent) to the slotted components and cure in a 65 °C oven overnight.

## 7 Operation Instructions

**Safety Tip:** It is recommended to wear cut-resistant gloves when handling the sharp blade during assembly and operation.

**Specimen preparation:**

1. **Assemble the slicer components**: First, assemble the shell and box.
2. **Prepare the specimen:** Add a 1–2 mL layer of agarose into the box, followed by placing the organoids inside. Fill the rest of the box with additional agarose.
3. **Solidify the agarose:** Place the box on ice to allow the agarose to solidify. Once the agarose is solid, remove the shell. No adhesives like superglue are needed, as the solidified agarose and box structure will securely hold the organoids in place. The box is positioned by sliding it into the shelves.
4. **Note on liquid usage:** Unlike traditional vibratomes, the specimen does not need to be submerged in liquid during slicing. Additionally, there is no need to collect each slice immediately, as the slices remain embedded within the agarose block, allowing multiple slices to be produced in succession. Given that only a few slices are typically obtained at a time, it is not necessary to keep the specimen cold. Unlike vibratomes, which require extended time and continuous cooling due to the large number of sections produced, our device slices within 5–10 min. The agarose is prepared with culture media, ensuring the tissue remains viable throughout the process without the need for cooling or submersion in media. However, after each slice is released from the agarose, it should be placed in DPBS to maintain viability.

**Step-by-step slicing procedure:**

1. Ensure the specimen is loaded into the box and stable.
2. Raise and stabilize the cutter before proceeding:
  a. Use the crank handle to rotate the main wheel, lifting the razor to its highest point.
  b. Insert the support screw through one of the top holes in the slotted supports.
  c. Gently allow the cart to rest on the support screw, then release the system.
3. Slide the box under the shelves, ensuring it is oriented as shown in Fig. 1.
4. Use the crank handle to raise the razor to its highest point, then hold it there while removing the support screw.
5. Lower the razor until it just touches the top of the specimen (without cutting it), and use the motion stage knob to align the razor with the desired cutting location.
6. Raise the razor back to its highest point with the crank handle, then rotate it one full loop to slice the specimen.
7. After completing a full loop, stabilize the cutter by inserting the support screw to hold the razor above the specimen.
8. Adjust the motion stage knob to set the thickness of the next cut.
9. Lift the razor, remove the support screw, and make another full loop to slice again.
10. After slicing is complete, stabilize the cutter, then gently slide the box out from the shelves to extract your specimen.

**Settings for determining thickness:**

The motion stage is measured in inches and can move a total of 0.5 in. in the x and y direction. One full rotation on the x knob is equivalent to 1/40 of an inch. *Example:* If you’re desired slice thickness is 1.2 mm then the knob needs to be turned twice in the x direction.

**Settings for rotational speed:**

It is recommended to operate the slicer at 1 rotation/5 s. Our characterization of the precision and straightness of the slicing was done at 1 rotation/5 s. Operating at this speed ensured that the slices were both straight and precisely cut the desired thickness each time it was operated. A quick experiment, operating the slicer at 1 rotation/1 s lead to less precise cuts where the target thickness being 0.75 mm and the slice resulted in 0.55 mm. Users should use the recommend settings to minimize variability.

## 8 Validation and characterization

A relevant use case for this particular hardware would be slicing a centre piece out of an agarose embedded live brain organoid with precision and accuracy. In typical organoids culture, significant necrosis and non-functional cells are often found in the core of organoids due to the diffusion limit of oxygen and nutrients. Recent studies have demonstrated that sliced brain organoids show improved cell viability and functional activities [4]. Therefore, here we will use slicing brain organoids as an example to characterize the performance of our device in terms of slicing precision, cut straightness, and cell viability after slicing.

### 8.1 Precision of the slice thickness

**Fig. 5:**
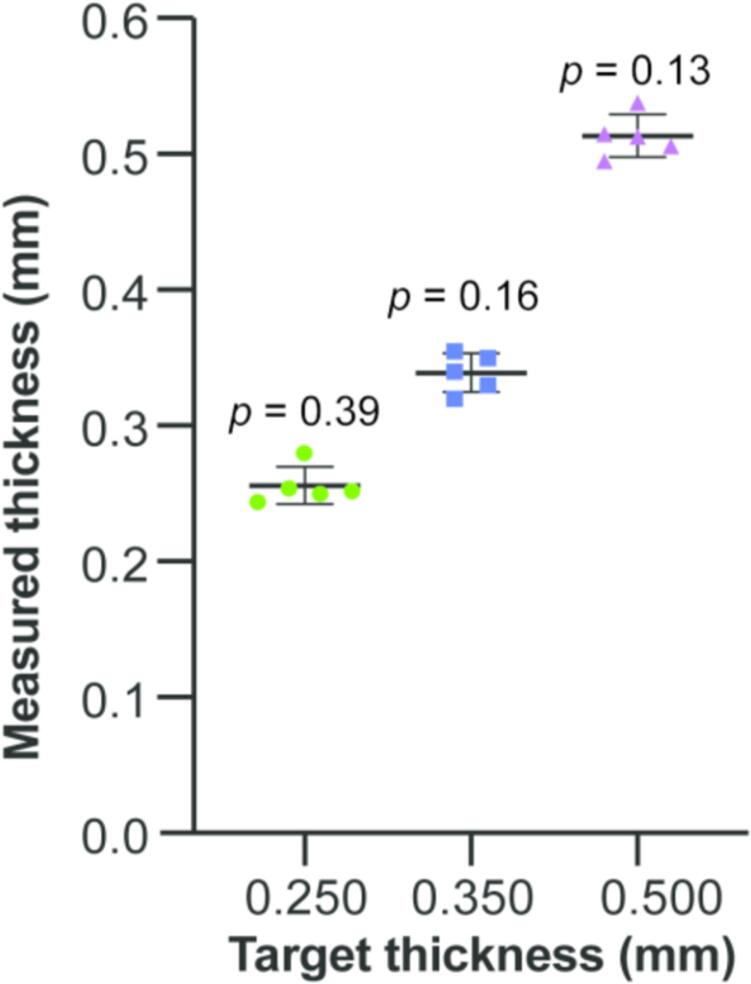
The precision of slice thickness. Dot plots shown targeted thickness and actual slice thickness (n = 5 for each thickness). Error bars indicate standard error of mean, and *p* values are calculated using one-sample student *t*-test.

We first tested the precision of the slice thickness by comparing the actual slice thickness with targeted thickness. Agarose gel block was sectioned at three distinct target thicknesses (0.250 mm, 0.350 mm, and 0.500 mm, respectively), which were typically used in slicing organoids considering diffusion limits. Slices aimed at a thickness of 0.250 mm, averaged a measurement of 0.256 ± 0.006 mm. 0.350 mm slices yielded an average thickness of 0.339 ± 0.006 mm. For 0.500 mm slices, the average thickness was determined to be 0.513 ± 0.007 mm. No statistical significance between the targeted value and the slices sectioned were found for all three thicknesses (Fig. 5).

### 8.2 Cut straightness Testing

We measured cut straightness through the agarose by characterizing the z-axis deflection. We took a macro shot video of the acrylic box with agarose stabilized. By imaging analysis, we were able to model scaled axes (in mm) to the macro shot video and measure the deflection from a vertical line from the base. We then noted the z-axis deflection at 1, 2, 3, 4 and 5 mm above the box base by pausing the video at each mm above the box base. We found an average z-axis deflection of Δz=0.115±0.015mm and no statistical significance was found when compared to perfectly straight slice, or Δz=0, (*p* = 0.070, one-sample student *t*-test).

### 8.3 Organoid viability tests

**Fig. 6:**
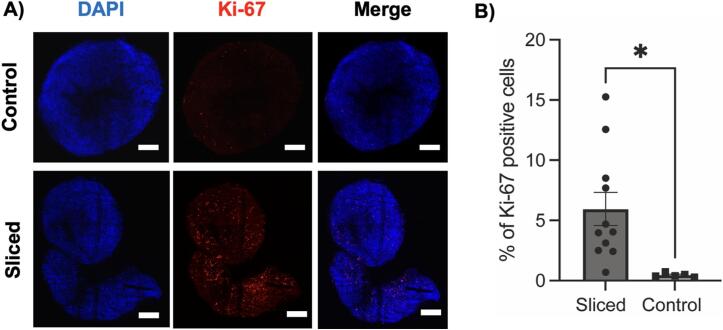
**Immunostaining of sliced organoids showing improved cell proliferation**. A) Representative confocal microscopy images showing DAPI staining (blue) indicating cell nuclei and red fluorescence indicating Ki-67 positive cells in sliced and control organoids. B) Bar plot quantification of Ki-67 positive cells. Error bars indicate standard error of mean, and *p* values are calculated using two-sample student *t*-test. Scale bar, ∼200um. *, *p < 0.05*. (For interpretation of the references to colour in this figure legend, the reader is referred to the web version of this article.)

To assess the viability of organoids post-slicing, we quantified the ratio of proliferating cells to total cells. Brain organoids were derived from human embryonic stem cells using a previously published protocol (*6*). Organoid slicing was conducted on day 153 of organoid maturation, followed by a five-day post-slicing culture period. Subsequently, the slices were fixed and stained with Ki-67 antibody to visualize cell proliferation, alongside DAPI staining. Quantification of Ki-67 and DAPI signals using Nikon Elements −GA3 software revealed a significantly higher percentage of Ki-67 + cells in sliced organoids compared to the unsliced controls (Fig. 6). This suggests that sliced organoids are actively proliferating, indicating that slicing does not compromise their viability, and improved nutrient and oxygen diffusion facilitate the development of organoids.

## 9 Discussion

Organoids that were not sliced exhibited sparse Ki67 detection, whereas those that were sliced showed an abundance of Ki67-positive cells. In our study, organoids sliced at day 153 exhibited Ki67 positivity in 5–15 % of the total cell population, compared to less than 3 % in control unsliced organoids. A previous study using a Leica vibratome to slice organoids at day 150 reported similar trends, with an abundance of Ki67-positive cells in the progenitor zones at approximately 15 %, while unsliced organoids showed sparse Ki67 presence in the progenitor zones, around 5 % [5]. While that study focused specifically on progenitor zones and ours on the entire organoid, the overall values and trends are comparable. The large variability between the sliced and control organoids is expected due to intrinsic heterogeneity of neural organoids and random distribution of proliferating neural progenitor cells [6].

While our low-cost slicer has been shown to effectively section brain organoids in hydrogels with minimal impact on cell viability, there are some limitations when translating this technology to other tissue types, such as brain, tumor, or lymph node explants. For softer tissues, like actual brain tissue, the absence of vibration in our slicer could lead to less precise cuts and increased tissue damage, as these tissues are more delicate compared to the agarose gel used in our experiments. Additionally, our system requires tissue embedding in agarose, which may compromise cell viability in certain tissues, particularly if longer slicing times are needed. However, in cases where gel embedding is feasible and only a few slices are required, our slicer could still provide a useful, low-cost alternative for tissue sectioning.

While this alternative is cost-effective, it does have certain limitations. Unlike vibratomes, the organoids are not immersed in a buffer or maintained in a O_2_-enriched environment, requiring slices to be made quickly to preserve viability. Additionally, the manual cranking mechanism introduces variability in the applied force, potentially leading to inconsistent results across users. To address these limitations, automation of the device would be a beneficial improvement. Integrating an image recognition system to detect the organoid and automatically control the stage for precise slicing could standardize results and enhance reproducibility. Furthermore, enlarging the sample chamber to accommodate a buffer and O_2_, as is done with vibratomes, would improve organoid viability over longer periods.

Declaration of Generative AI and AI-assisted technologies in the writing process

During the preparation of this work the author(s) used ChatGPT in order to correct for grammar and punctation. After using this tool/service, the author(s) reviewed and edited the content as needed and take(s) full responsibility for the content of the publication.

## CRediT authorship contribution statement

**Beatriz Martinez-Martin:** Writing – original draft, Validation, Formal analysis, Data curation, Conceptualization. **Isabella Lambros:** Writing – original draft, Methodology, Data curation, Conceptualization. **Lukas Nuesslein:** Data curation. **Yubing Sun:** Writing – review & editing, Supervision, Conceptualization.

## Declaration of competing interest

The authors declare that they have no known competing financial interests or personal relationships that could have appeared to influence the work reported in this paper.
